# Development of Triamcinolone Acetonide Nanocrystals for Ocular Administration

**DOI:** 10.3390/pharmaceutics15020683

**Published:** 2023-02-17

**Authors:** María Lina Formica, Hamoudi Ghassan Awde Alfonso, Alejandro Javier Paredes, María Elisa Melian, Nahuel Matías Camacho, Ricardo Faccio, Luis Ignacio Tártara, Santiago Daniel Palma

**Affiliations:** 1 Unidad de Investigación y Desarrollo en Tecnología Farmacéutica (UNITEFA), CONICET and Departamento de Ciencias Farmacéuticas, Facultad de Ciencias Químicas, Universidad Nacional de Córdoba, Ciudad Universitaria, Córdoba 5000, Argentina; 2 School of Pharmacy, Queen’s University Belfast, 97 Lisburn Road, Belfast BT9 7BL, UK; 3 Área de Farmacología, Departamento de Ciencias Farmacéuticas—CIENFAR, Facultad de Química, Universidad de la República (Udelar), Av. General Flores 2124, Montevideo 11800, Uruguay; 4 Área Física, Departamento de Experimentación y Teoría de la Estructura de la Materia y sus Aplicaciones—DETEMA, Facultad de Química, Universidad de la República (Udelar), Av. General Flores 2124, Montevideo 11800, Uruguay

**Keywords:** triamcinolone acetonide, nanocrystals, ocular inflammation, corticosteroids, media milling

## Abstract

Triamcinolone acetonide (TA) is a powerful anti-inflammatory drug used in the treatment of inflammatory ocular disorders; however, its poor aqueous solubility and ocular anatomical barriers hinder optimal treatment. The aim of this work was to obtain triamcinolone acetonide nanocrystals (TA-NC) to improve ocular corticosteroid therapy. Self-dispersible TA-NC were prepared by the bead milling technique followed by spray-drying, exhaustively characterized and then evaluated in vivo in an ocular model of endotoxin-induced uveitis (EIU). Self-dispersible TA-NC presented an average particle size of 257 ± 30 nm, a narrow size distribution and a zeta potential of −25 ± 3 mV, which remained unchanged for 120 days under storage conditions at 25 °C. In addition, SEM studies of the TA-NC showed uniform and spherical morphology, and FTIR and XRDP analyses indicated no apparent chemical and crystallinity changes. The subconjunctival administration of TA-NC in albino male white rabbits showed no clinical signs of ocular damage. In vivo studies proved that treatment with self-dispersible TA-NC alleviated the inflammatory response in the anterior chamber and iris in EUI rabbit eyes. Dispersible TA-NC are a promising approach to obtaining a novel nanometric TA formulation for ocular disorders.

## 1. Introduction

Corticosteroids are widely used drugs in the treatment of several inflammatory ocular disorders, such as diabetic macular edema (DME), retinal vein occlusion, age-related macular degeneration (AMD) and uveitis [1]. Corticosteroids act by inhibiting inflammation primarily through interaction with glucocorticoid receptors. Although these drugs are considered effective, the duration of their therapeutic effect is conditioned by their pharmaceutical dosage form, the route of administration and their potential side effects. Topical ocular administration is the route of choice for patients; however, less than 5% of the instilled drug is usually absorbed in the ocular tissue, and a minimal amount usually reaches the posterior segment, limiting the treatment of posterior segment pathologies. On the other hand, systemic administration of corticosteroids requires high doses to achieve therapeutic levels of the drug in the eye, which can be accompanied by severe systemic side effects [2].

In this way, the intravitreal administration of corticosteroids as a depot of drug suspensions is usually used to allow effective concentrations in the back of the eye. However, in chronic pathologies, frequent injections are required, which are associated with risks of retinal detachment, endophthalmitis and intravitreal haemorrhage. In addition, chronic treatment with corticosteroids may lead to increased intraocular pressure and predispose to the development of cataracts. A biodegradable intravitreal implant loaded with a corticosteroid has been developed to overcome these drawbacks and allow sustained drug release. Nevertheless, it presented some limitations associated with its administration technique and high cost [3].

Among other corticosteroid therapies, ocular injections of triamcinolone acetonide (TA) are used as an effective and low-cost treatment for different ocular diseases [4,5]. Topical administration of TA to the inner part of the eye is problematic due to ocular barriers and drug properties such as low solubility in body fluids and limited permeability [6]. However, intravitreal or periocular administration of TA increases its delivery to the vitreous cavity, providing prolonged drug action related to a depot effect [7,8]. Thus, the available FDA-approved formulations of TA are applied intravitreally, such as Triescence™-Alcon Research, Ltd. and Trivaris™-Allergan, Inc. Mainly in developing countries, other formulations based on the intravenous suspension of TA are still administered by ocular routes as off-label drugs for the treatment of inflammatory processes of ocular diseases such as DME, uveitis and ADM, among others. Although injectable TA suspension is widely used by clinical ophthalmologists, it presents certain limitations associated with its particle size and preservatives in its formulation, which may hinder its administration and cause ocular discomfort [9]. Therefore, the development of new therapeutic strategies of TA for ocular application is interesting due to its proven efficacy, low cost and the broad range of pathologies for which it is prescribed. Moreover, the limitations related to its route of administration, inclusion in pharmaceutical forms and the need of an effective ocular delivery, must be resolved.

Ocular delivery systems based on nanotechnology have proven to be effective and safe for ocular drug delivery. Different nanotechnological strategies have been developed to obtain TA delivery systems for ocular applications such as liposomes [10], lipid nanocapsules [11], nanostructured lipid carriers [12], microneedles [13] and nanoemulsions [14]. Beyond the fact that these strategies have shown promising results for the ocular delivery of TA, the availability of simpler formulations that allow TA to be maintained as a low-cost therapeutic option is much needed. Hence, the formulation of nanocrystals (NCs) is an interesting strategy to obtain simple nanotechnology-based pharmaceutical formulations that can be prepared by simple and scalable processes.

Drug NCs are solid particles of a drug of nanometric size, typically between 200 and 500 nm [15], surrounded by a stabilizing layer. Usually, stabilizers are amphiphilic surfactants or polymers. NCs can be produced from nanosuspensions (NSs) of drugs, in which nanometric particles of the drug are dispersed in an aqueous medium that can be removed to obtain the NCs as solid nanoparticles.

NCs provide a higher drug dissolution rate, saturation solubility and adhesiveness [16,17]. These physicochemical features allow for improved bioavailability and therapeutic efficacy [18]. Our research group has been working on the development of NC formulations that demonstrate improved biopharmaceutical and pharmacokinetic behaviour of poorly water-soluble drugs via several administration routes [19,20]. Taking into account the above considerations on the need for the development of TA formulations and the advantages of NCs, this work aimed at the development of triamcinolone acetonide nanocrystals (TA-NC) as a novel therapeutic strategy for ocular TA administration. Thus, self-dispersible TA-NC were prepared by media milling followed by spray-drying. Exhaustive physicochemical analyses were performed to characterize the NC, and in vivo studies using albino rabbits were carried out to evaluate the therapeutic efficacy of TA-NC compared to an injectable suspension commonly used off-label for ocular application.

## 2. Materials and Methods

### 2.1. Materials and Reagents

The micronized form of triamcinolone acetonide (mTA) with a purity of 99.5% was purchased from Pura Quimica^®^ (Córdoba, Argentina). Poloxamer 188 (P188) was provided by Rupamel S.R.L (Buenos Aires, Argentina), a sales agent of BASF. Ultrapure water was obtained from Water Purification System (HF-Super Easy Series, Heal Force, Shanghai, China). Escherichia coli lipopolysaccharide (LPS) was purchased from Sigma-Aldrich^®^. Zirmil^®^ Yttrium-stabilized zirconium beads of 0.1 mm size (Saint-Gobain ZirPro, Köln, Germany) were used as a collision agent in the milling process. All other chemicals were extra pure grade and used without further purification.

### 2.2. Preparation of Triamcinolone Acetonide Nanosuspensions (TA-NS)

Triamcinolone acetonide nanosuspensions (TA-NS) were prepared by the wet bead milling (WBM) technique as described previously [19] using a NanoDisp^®^ laboratory-scale mill (NanoDisp^®^, Córdoba, Argentina). Briefly, mixtures of mTA and P188 were homogenized in a porcelain mortar for 5 min and ultrapure water was added gradually up to 100 mL to form suspensions. The drug suspensions and 0.1 mm zirconia beads (25% *v*/*v*) were then placed in the milling chamber and processed at 1600 rpm for 120 min at a fixed temperature of 15 °C by the circulating cold water with a Thermo Haake^®^ compact refrigerated circulator (Thermo Fisher Scientific, Waltham, MA, USA). The processed samples were evaluated in terms of average particle size (APS) and polydispersity index (PDI) every 30 min. An experimental mixture design was carried out to evaluate the influence of the component ratio of the TA-NS on the colloidal properties. Table 1 shows the ratio of mTA and P188 in the experimental mixture design formulations with a total solids content of 2% *w*/*v* (NS1-NS5). In turn, wet milling of a TA-NS with a total solid content of 6% *w*/*v* (NS6) composed of TA and P188 with a mass ratio of 1:1 was prepared by WBM with the same process parameters to evaluate the colloidal properties at a higher solids concentration.

### 2.3. Preparation of Dried Dispersible Triamcinolone Acetonide Nanocrystals

After the WBM process, the water in the TA-NS was immediately removed by spray-drying using a Büchi B-290 mini spray-drier (Büchi Labortechnik AG, Flawil, Switzerland) equipped with a dehumidifier module, using a two-fluid nozzle with a cap orifice diameter of 1.5 mm. The drying process was carried out with the following set conditions: inlet temperature of 45 °C, atomizing air flow rate of 600 L/h, pump speed of 2 mL/min and aspiration at 30 m^3^/h. In addition, slow magnetic stirring was kept constant during the drying process. The dried powders were weighed to determine the process yield and moisture and stored at 4–8 °C and room temperature (25 °C) under dry conditions.

### 2.4. Process Yield and Moisture Content

The process yield percentage (*PY%*) after water removal was calculated by the following equation:

(1) $$PY\;\left(\%\right)=\frac{A}{B}\times 100$$

where *A* is the solids weighed of TA-NC recovered after drying and *B* is theoretical initial solids (mTA and P188 weighed), considering the initial dry mixture of powders (drug and P188) as the theoretical 100%.

The moisture of the dried redispersible TA-NC was measured immediately after the removal of water using a halogen heating autoanalyzer (Ohaus M45^®^, Greifensee, Switzerland) at 90 °C.

### 2.5. Physical Mixture Preparation

A physical mixture (PM) between broken granules of P188 and mTA was prepared as a control for physicochemical characterization in a mass ratio of 1:1 of both components. Briefly, mTA and P188 were mixed and ground in a porcelain mortar.

### 2.6. Physicochemical Characterization of NC-TA

Samples from the milling process, TA-NS and dried TA-NC, were analyzed in terms of APS, PDI and zeta potential (ZP) using a Zetasizer NanoSerie DTS 1060 (Malvern Instruments S.A., Worcestershire, UK) at 25 °C in triplicate. Previously, a small fraction (spatula tip) of the dried TA-NC was dispersed in 5 mL of ultrapure water at room temperature. In addition, dried powders were stored at room temperature (~25 °C), while the redispersed TA-NC nanosuspensions were stored at both room temperature (~25 °C) and 4 °C for 30 days. The following parameters were evaluated at different time points: macroscopic aspects (aggregate formation and colour changes), APS, PDI and ZP.

### 2.7. Scanning Electron Microscopy (SEM)

Images of mTA, PM and dried TA-NC were taken with FE-SEM Σigma (ZEISS, Oberkochen, Germany). Powdered samples were deposited in an aluminum well and sprayed with chromium prior to morphological analysis. Magnification ranges between 500 and 10,000× were used.

### 2.8. Fourier Transform Infrared Spectroscopy (FTIR)

Powdered samples of mTA, PM, P188 and dried TA-NC were analyzed by FTIR using a Nicolet FTIR 5-SXC^®^ Infrared Spectrophotometer. Four clean-up scans were performed with a threshold of 0.002; 40 background signal scans were made in a spectral range of 650–4000 cm^−1^ and with a deuterated triglycine sulfate detector.

### 2.9. Differential Scanning Calorimetry (DCS) and Thermogravimetry (TGA)

For DSC analysis, powdered samples of mTA, PM and TA-NC were deposited in aluminum pans and examined at a heating rate of 10 °C/min in the temperature range between 10 and 305 °C using a Discovery DSC 25P instrument (TA Instruments, New Castle, DE, USA) under a dynamic N_2_ atmosphere (50 mL/min). The DSC cell was calibrated with indium (mp 156.6 °C; ΔHfus = 28.54 J/g). TGA analysis was performed under the same established conditions in the temperature range of 10–350 °C and a dynamic N_2_ atmosphere (50 mL/min) at a heating rate of 10 °C/min using a TGA instrument (Discovery HP TGA, TA Instruments, New Castle, DE, USA).

### 2.10. X-ray Powder Diffraction (XRPD)

Powder samples of mTA, P188, PM and TA-NC were examined in terms of crystallinity using a Rigaku Miniflex X-ray powder diffractometer (Rigaku, Tokyo, Japan) with CuKα radiation (λ = 1.5418 Å) operating at 40 kV, 15 mA and utilizing a D/tex Ultra2 1D detector. Measurements were carried out in the scan mode over a 2θ range from 2° to 60° with a step size of 2θ = 0.01° and a speed of 5°/min.

### 2.11. Confocal Raman Microscopy

Confocal Raman microscopy was performed using WITec Alpha 300-RA confocal Raman microscopy equipment (WITec, Ulm, Germany). The excitation laser wavelength corresponded to λ = 532 nm and the nominal power was adjusted to 45 mW to avoid sample decomposition. Raman spectra of the pure components were obtained by averaging a set of 100 spectra with an integration time of 0.512 s for each spectrum. Two-dimensional confocal Raman microscopy images of the PM and TA-NC samples were collected at random locations of 150 × 150 µm areas with 50 × 50 point grids defining the bitmap image (2500 pixels). Individual Raman spectra were collected for each pixel in the selected areas with an integration time of 0.112 s. The spectrometer operating with 600 lines/mm grating allowed us to obtain spectra with a resolution of ~4 cm^−1^ in the range of 70–4000 cm^−1^. All images were collected at an optical resolution limit of ~300 nm. True component analysis was used to map the spatial distribution of mTA and P188 in the PM or TA-NC samples using ProjectFive 5.1 Plus software (WITec, Ulm, Germany).

### 2.12. Drug Saturation Concentration

Excess amounts of mTA, PM and dried dispersible TA-NC were added to tubes containing 1 mL of simulated tear fluid (pH = 7.4) and they were placed in a water bath and shaken at 37 °C. Afterward, they were centrifuged (2990× *g*, 5 min and 37 °C) and the supernatants were collected and filtered (0.45 µm PVDF membrane). Then, drug concentration was determined by U-Vis. Different time intervals (0.25, 0.5, 0.75, 1, 2, 4, 6, 24, 48 and 72 h) were evaluated in triplicate.

### 2.13. Animals

Male albino white rabbits (weight, 2.0–2.5 kg) were treated according to the guidelines of the ARVO Statement for the Use of Animals in Ophthalmic and Vision Research. Every effort was made to reduce the number of animals used. All experimental procedures were approved by the Institutional Animal Care and Use Committee (CICUAL) of the Facultad de Medicina, Universidad Nacional de Córdoba (School of Medical Sciences, National University of Cordoba) (No. 44/2017).

### 2.14. Ocular Tolerance and Irritation Test

Ocular irritation tests were performed using male albino white rabbits (*n* = 10). Five rabbits were injected subconjunctivally with a dispersible TA-NC suspension (40 mg/mL, 50 μL) in the right eyes, while 50 μL of normal saline solution (NSS) was applied as a control in the left eyes. Fiver other rabbits were subconjunctivally injected with 50 μL of Fortcinolona^®^ 40 suspension (40 mg/mL) in the right eyes, while the left eyes were observed as normal eyes. Clinical signs of damage in the anterior and posterior segments in response to treatments were evaluated using a slit lamp (Huvitz HIS5000, Anyang-si, Gyeonggi-do, Republic of Korea) and indirect ophthalmoscopy by an experienced ophthalmologist. Furthermore, the integrity of the corneal epithelium was checked by fluorescein staining.

### 2.15. Endotoxin-Induced Uveitis (EIU)

For the preclinical evaluation of TA-NC, we used an EIU rabbit model based on others reported in the literature [21,22], which was previously optimized and adapted in our laboratory [11]. All rabbits were topically anesthetized with 0.5% proparacaine hydrochloride ophthalmic solution and then injected with 60 μL of LPS (Sigma-Aldrich^®^, Darmstadt, Germany) 1 ng/μL via the intravitreal route using a 30G needle. After 1 h of model induction, the groups of animals were injected subconjunctivally (50 μL) with a single dose as follows: I) a dispersible TA-NC suspension (40 mg/mL) (*n* = 8), II) Fortcinolona^®^ 40 (40 mg/mL) (*n* = 8) and III) NSS, the control group of the EUI model (*n* = 8). The clinical signs of the different groups were compared with IV) normal animals (*n* = 8) that were neither subjected to the EUI model nor given any treatment. Before determining the clinical signs of different groups, 0.5% topical tropicamide (Alcon Mydril™-Alcon Inc., Fort Worth, TX, USA) and topical anesthesia (0.5% proparacaine hydrochloride ophthalmic solution) were applied to all animals. Two masked experienced ophthalmologists examined the animals by slit-lamp and binocular indirect ophthalmoscopy (Heine small pupil) using a 20D lens (VOLK, Mentor, OH, USA) after 24, 48 and 72 h of the EIU model induction. A clinical inflammation scoring criterion was used to assess the severity of EIU, which took into account the anterior chamber flare, Tyndall effect by anterior chamber cells, fibrine deposits and iris vessel congestion. The anterior chamber flare and Tyndall effect of anterior chamber cells were valued between 0 and 8, while the other parameters were scored between 0 and 4 for each parameter.

Moreover, the aqueous humour fogging in the anterior chamber (flare) was quantified using ImageJ^®^ software (64-bit, Java 8) from ocular photographs taken with Canon EOS Rebel T6 coupled to the slit lamp and calculated as the difference between the mean grey of the corneal light reflection and the anterior camera in each group of animals. An optically cut section of the eye was optically obtained from a very thin parallelepiped slice (3 × 3 mm) of the cornea with a slit lamp microscope (16×) using a 45-degree angle between the illumination and viewing paths. Then, the aqueous humour transparency percentage (AHT%) in the anterior chamber was calculated by the following equation, considering that this difference from mean grey in normal rabbits corresponds to 100%:

(2) $$AHT\%=\frac{D}{E}\times 100$$

where *D* is the aqueous humour fogging of animals after induction of the EIU model and *E* is the aqueous humour fogging of normal rabbits (baseline response). In addition, the intraocular pressure (IOP) was measured with a calibrated digital ICARe^®^ tonometer.

### 2.16. Statistical Analysis

For each parameter, ANOVA analysis was performed using Minitab^®^18 software or Prism-GraphPad, together with the Tukey pairwise comparison, and both ANOVA and Tukey’s mean comparison analysis results were expressed as mean values ± standard deviation (SD). *p* < 0.05 was considered to be statistically significant.

## 3. Results

### 3.1. Preparation of TA-NS and Dispersible TA-NC

In order to obtain self-dispersible TA-NC, the preparation of TA-NS was first studied. TA-NS were successfully obtained by the WBM technique with the set processing conditions. In order to evaluate the influence of the component ratio of mTA and P188 on the colloidal properties and stability, TA-NS were prepared with different ratios of drug and stabilizer between 0.250 and 1.725%. The results of the experimental mixture design for the preparation of TA-NS (NS1-NS5) with a total solids content of 2% *w*/*v* are shown in Table 1.

**Table 1:** Composition and colloidal parameters of triamcinolone acetonide nanosuspensions (TA-NS) and self-dispersible triamcinolone acetonide nanocrystals (TA-NC) obtained by bead milling and spray-drying, respectively.

	Solid Components of TA-NS			TA-NS after Wet Milling			Dispersed TA-NC after Spray-Drying					
TA-NS	TA (%)	P188 (%)	TSC (%)	Size (nm)	PDI	ZP (mV)	TA-NC	Size (nm)	PDI	ZP (mV)	PY %	MC%
NS1	1.750	0.250	2	250.5 ± 3.2	0.14 ± 0.03	−30.6 ± 0.7	NC1	757.8 ± 13.7	0.52 ± 0.10	−21.0 ± 0.7	51.9	1.28
NS2	1.375	0.625	2	305.3 ± 7.8	0.15 ± 0.03	−25.1 ± 0.5	NC2	326.6 ± 4.2	0.260 ± 0.003	−24.8 ± 0.2	62.2	1.38
NS3	1	1	2	231.8 ± 2.9	0.12 ± 0.02	−25.9 ± 0.3	NC3	260.1 ± 1.6	0.18 ± 0.09	−26.1 ± 0.4	69.2	1.18
NS4	0.625	1.375	2	234.8 ± 2.0	0.12 ± 0.02	−27.2 ± 0.3	NC4	260.1 ± 2.5	0.15 ± 0.02	−25.2 ± 0.9	69.5	1.16
NS5	0.250	1.750	2	312.9 ± 8.3	0.21 ± 0.02	−32.8 ± 0.1	NC5	232.3 ± 1.2	0.14 ± 0.05	−29.9 ± 0.6	68.5	1.57
NS6	3	3	6	274.7 ± 45.8	0.15 ± 0.05	−28.3 ± 3.4	NC6	257.3 ± 30.5	0.15 ± 0.08	−24.9 ± 2.9	60.0	1.26

Abbreviations: APS: Average particle size; MC%: Moisture content percentage; PDI: Polydispersity index; PY%: Process yield percentage; TSC: Total solids content; ZP: Zeta potential.

**Figure 1:**
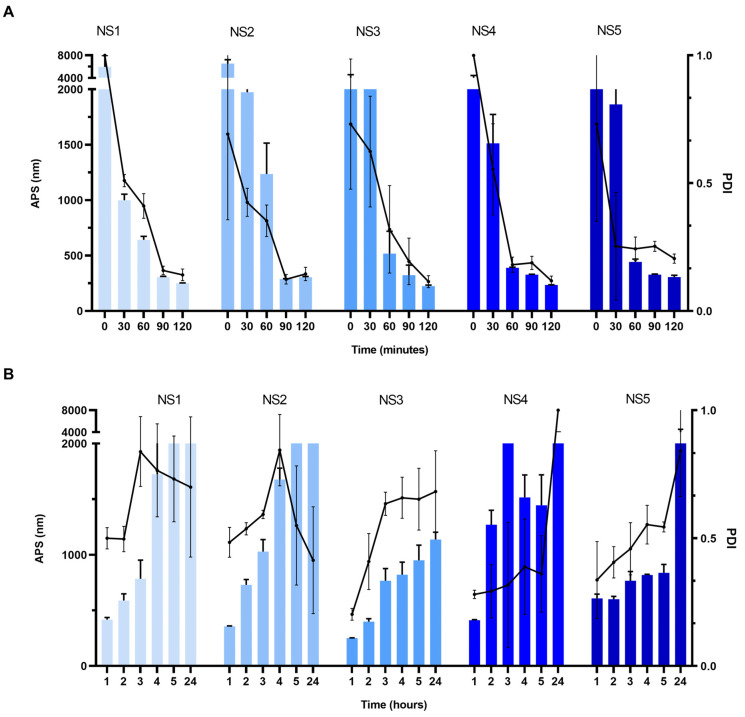
Average particle size (APS) and polydispersity index (PDI) of different drug nanosuspension from mixture design (**A**) during the wet milling process and (**B**) after the wet milling process at room temperature. Results are presented as means ± SD (*n* = 3) for all tested drug nanosuspensions.

Despite the composition of the formulation tested, a decreasing trend in particle size and PDI was observed with increasing milling times as shown in Figure 1A. After 2 h of milling at the set parameters, all TA-NS formulations exhibited a particle size below 350 nm and a PDI below 0.2. A decrease in APS was observed in TA-NS with a high proportion of P188 and a low proportion of TA. NS3 and NS4 presented the lowest particle sizes, around 232 nm. Regarding PDI, it seems to decrease with increasing stabilizer concentration up to 1.375% as in NS1 to NS4, while it is the highest in NS5. All PZ values were negative, between −30 and −25 mV, appearing to increase in terms of absolute values with high stabilizer concentration.

Subsequently, the colloidal stability of different TA-NS under storage conditions at 25 °C was studied. As shown in Figure 1B, there is a progressive increase in the APS and PDI in all nanosuspensions, which doubled their value in the first 2 h and reached micrometric size after 24 h under this storage condition. Based on these results, water removal from TA-NS by the spray-drying process was carried out.

### 3.2. Dispersible TA-NC

Through the spray-drying process under the established parameters, different dried TA-NC (NC1, NC2, NC3, NC4 and NC5) were efficiently obtained from the respective TA-NS (NS1, NS2, NS3, NS4 and NS5). The spray-drying process yield of different TA-NS was higher than 50%, reaching values of 70% for TA-NC obtained from NS3, NS4 and NS5. Moreover, the moisture content of powder TA-NC was lower than 2%.

Table 1 shows the colloidal parameters of different dried TA-NC in terms of APS, PDI and ZP after dispersion in water. The dried TA-NC prepared with the TA-NS with a high proportion of P188 and a low proportion of TA showed lower APS and PDI and a higher negative ZP in terms of absolute value. Thus, the aqueous dispersed NC1 prepared from the TA-NS with only 0.250% of P188 showed an APS of 758 ± 14 nm and a high polydispersity (PDI = 0.52), presenting a large difference with the parameters of the starting nanosuspension (NS1). On the contrary, the NC5 prepared from the TA-NS with 1.725% of stabilizer exhibited an APS of 232 ± 1 nm and a narrow size distribution (PDI = 0.14), lower than that of the starting nanosuspension (NS5). Regarding the aqueous dispersed NC3 and NC4 prepared from TA-NS with stabilizer concentrations between 1.000 and 1.375% (NS3 and NS4, respectively), both showed an APS of 260 nm, slightly differing (around 30 nm) from the TA-NS (before the spray-drying process). Similarly, water-dispersed NC3 and NC4 exhibited a narrow size distribution, remaining with a PDI below 0.2 after the spray-drying process of NS3 and NS4.

**Figure 2:**
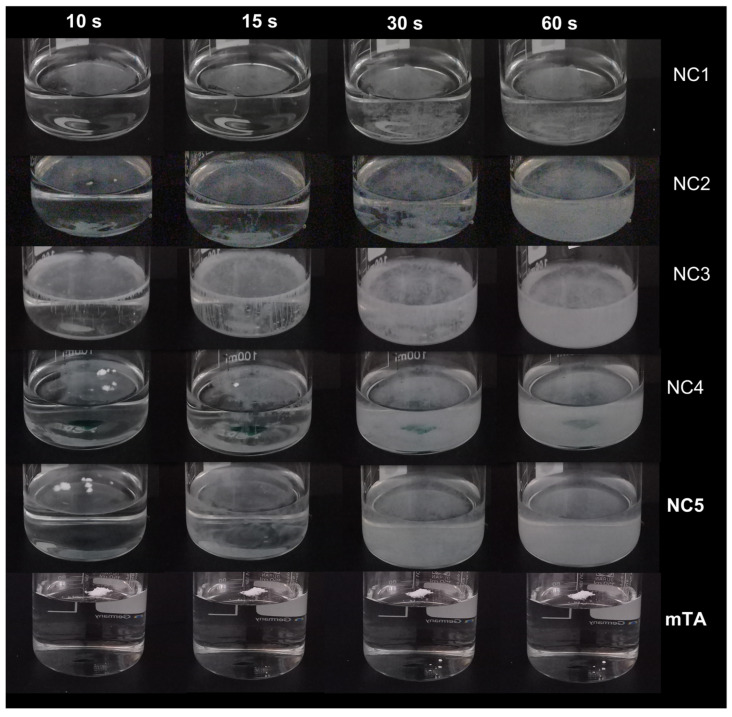
Illustrative images of the aqueous self-dispersion of the powdered TA-NC at 0, 15, 30 and 60 s.

Moreover, all dried TA-NC showed satisfactory aqueous self-dispersion, even those with a high proportion of drug and a low proportion of P188, while faster homogeneous self-dispersion occurs with an increasing amount of stabilizer (Figure 2). As a control, mTA dispersion in ultrapure water was tested under the same experimental conditions, which was not achieved. As illustrated in Figure 2, NC3 containing the same ratio of drug and stabilizer seems to exhibit the most homogeneous powder self-dispersion.

**Figure 3:**
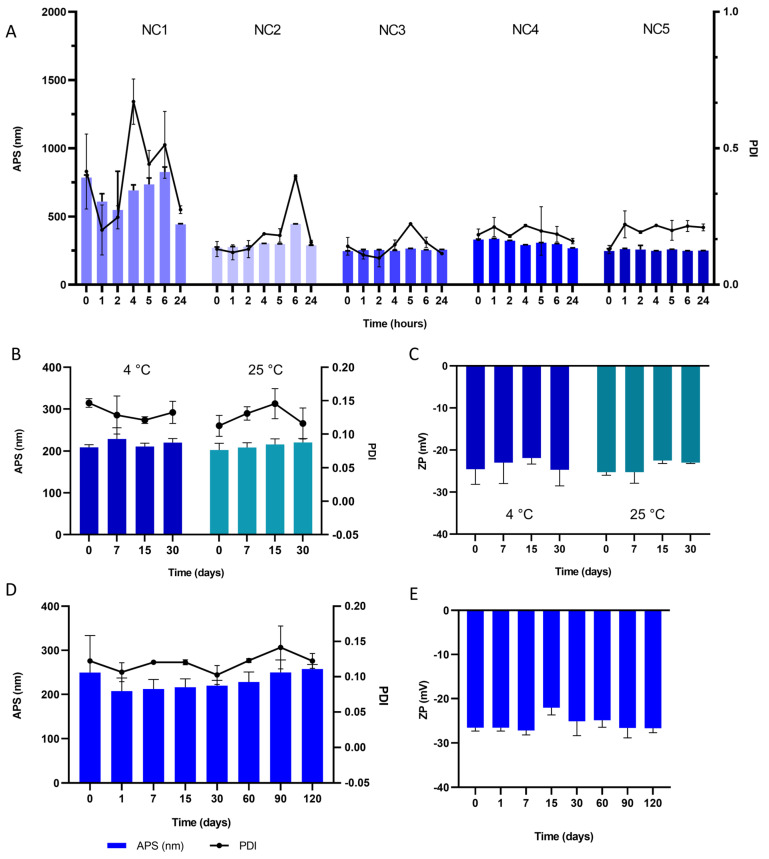
(**A**) Average particle size (APS, bar graph) and polydispersity index (PDI, line graph) of different TA-NC after dispersion in water. (**B**) APS and PDI, and (**C**) zeta potential (ZP) of nanosuspension from TA-NC (NC6) dispersed in water at different times under storage conditions at 4 °C and room temperature (~25 °C). (**D**) APS and (**E**) ZP of TA-NC powder (NC6) at different times under 25 °C storage conditions after water dispersion over time. Results are presented as means ± SD (*n* = 3) for all TA-NC.

Next, the stability of the different TA-NC stored at room temperature at different times was examined by measuring the particle size after dispersion in water (Figure 3). The TA-NC with the lowest stabilizer concentration exhibited a particle size difference of at least 100 nm between the different studied times after its dispersion in water. In contrast, no significant changes were observed in the APS of the TA-NC with high stabilizer concentration (NC3, NC4 and NC5) after 24 h of its aqueous dispersion. Considering the APS and PDI of TA-NS and dispersible TA-NC, colloidal stability and the use of minimum stabilizer concentration required in ocular pharmaceutical technologies, the TA-NC containing the same ratio of drug and stabilizer (1:1) was selected for further experiments.

In order to evaluate the preparation of TA-NC from a TA-NS with a higher total solids content (6%) by the described WBM process followed by spray-drying, a drug nanosuspension (NS6) composed of TA and P188 at a mass ratio of 1:1 was prepared. Compared to NC3, which had the same mass ratio with a lower total solids content, NC6 presented slight differences in terms of APS, PDI, ZP and moisture content. Moreover, the previous TA-NS (NS6) presented a comparable size reduction during the milling process resulting in nanoparticles around 275 nm and a PDI lower than 0.2, both slightly higher than those shown by NS3. Therefore, the preparation of TA-NC from the nanosuspension of the drug with a three-fold solids content is feasible at the tested concentrations of TA and P188 with a mass ratio of 1:1.

Subsequently, NC6 dispersed in ultrapure water and stored at 4 °C and 25 °C remained stable for thirty days in terms of APS, PDI and ZP under both storage conditions, showing no significant changes compared to initial values (Figure 3B,C). No macroscopic changes in appearance were observed at the end point of the study. For its part, the dry dispersible NC6 powder retained its APS for at least 120 days under storage at room temperature, as observed in Figure 3D,E.

The formulation corresponding to the dispersible TA-NC obtained from the TA-NS with TA and P188 with a mass ratio of 1:1 was selected to continue with further studies since it showed low APS, a narrow size distribution and low moisture content and was obtained with a high yield from the spray-drying process. In turn, previous TA-NS also exhibited low APS, a narrow size distribution and a lower rate of size increase under room temperature storage conditions after the milling process.

### 3.3. Scanning Electron Microscopy (SEM)

**Figure 4:**
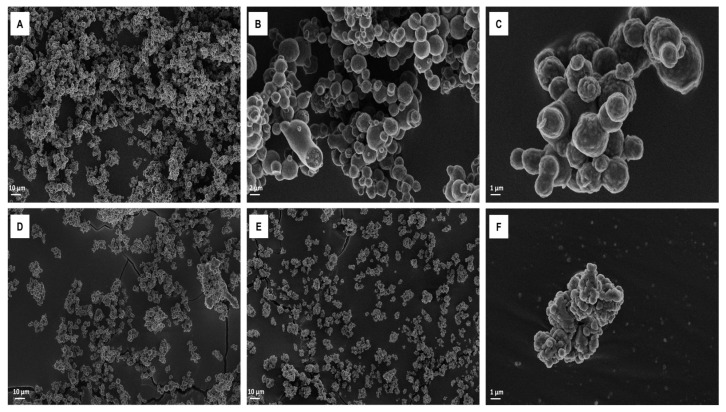
Scanning electron microscope at different magnifications of (**A**)TA-NC at 500×, (**B**) TA-NC at 3000× and (**C**) TA-NC at 10,000×, (**D**) PM at 500×, (**E**) mTA at 1000× and (**F**) mTA at 10,000×.

SEM study performed at 500× and 3000× magnification revealed that TA-NC are homogeneously distributed (Figure 4A) and presented spherical shapes with smooth surfaces (Figure 4B). Under 10,000×, TA-NC were observed to present a rougher surface and were distributed in microclusters of particle sizes around 1 µm (Figure 4C). In contrast to TA-NC, under 500× magnification, no distinct powder particles were observed in the mTA sample (Figure 4D), while at higher magnification (10,000×), particles distributed in heterogeneous macroclusters between 5 and 20 µm were observed (Figure 4B). In addition, PM showed heterogeneous aggregates of mTA and P188 between 5 and 50 µm (Figure 4F).

### 3.4. Fourier Transform Infrared Spectroscopy (FTIR)

**Figure 5:**
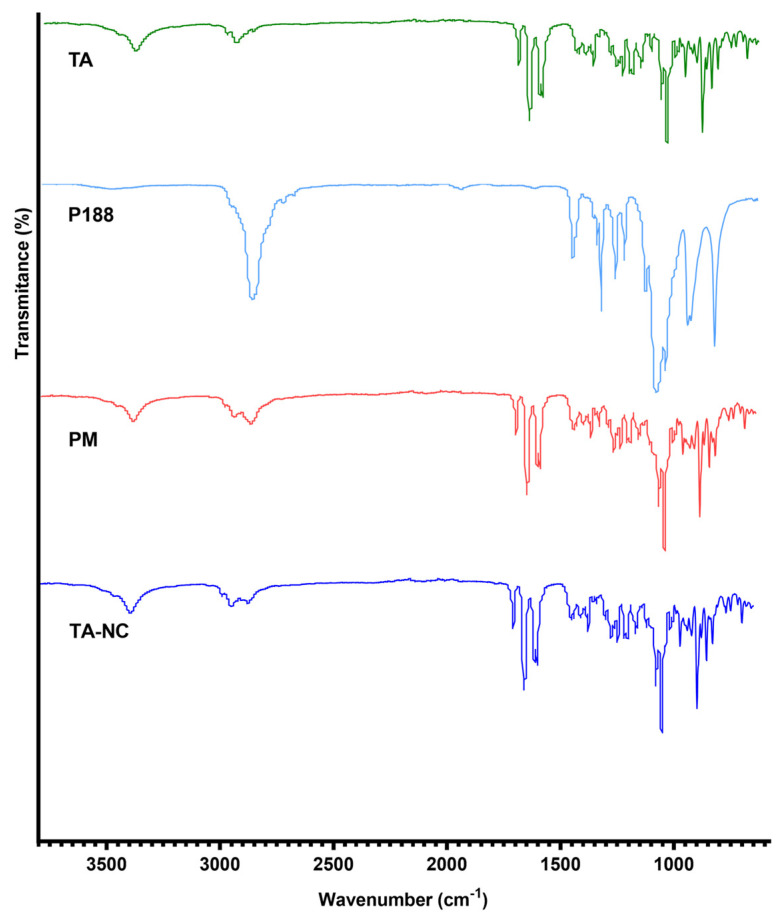
Fourier-transformed infrared (FTIR) spectrometry of mTA, P188, PM and TA-NC.

An FTIR spectrometry study was used to analyze the chemical interactions in the chemical structure of the TA-NC and whether there were chemical interactions between the drug and the stabilizer. Figure 5 shows that mTA and TA-NC presented typical infrared absorption bands around 3392 cm^−1^ associated with the O–H stretching vibration and around 1700 cm^−1^ related to the C–O aliphatic ketone present in the TA molecule. In turn, it showed a band at 2950 cm^−1^ that corresponds to the C–H vibrations. Moreover, in the FTIR spectra of mTA and TA-NC, characteristics peaks of TA were observed, such as bands around 1120 cm^−1^ corresponding to the asymmetric axial deformation of C–O–C bond in aliphatic esters and the peak around 1056 cm^−1^ corresponding to C–F stretching of the halogenated ring [13,23].

In addition, P188 showed absorption bands around 2900 cm^−1^, 1500 cm^−1^ and 1300 cm^−1^ corresponding to the aliphatic chains of the molecule, which were clearly observable in the FTIR spectrum of TA-NC, but with lower intensity. Furthermore, no substantial wavenumbers shifts or additional peaks were observed in the FTIR spectrum of TA-NC. All these results indicate that no detectable chemical interactions between TA and P188 occurred during the preparation process.

### 3.5. Differential Scanning Calorimetry (DCS) and Thermogravimetry (TGA)

**Figure 6:**
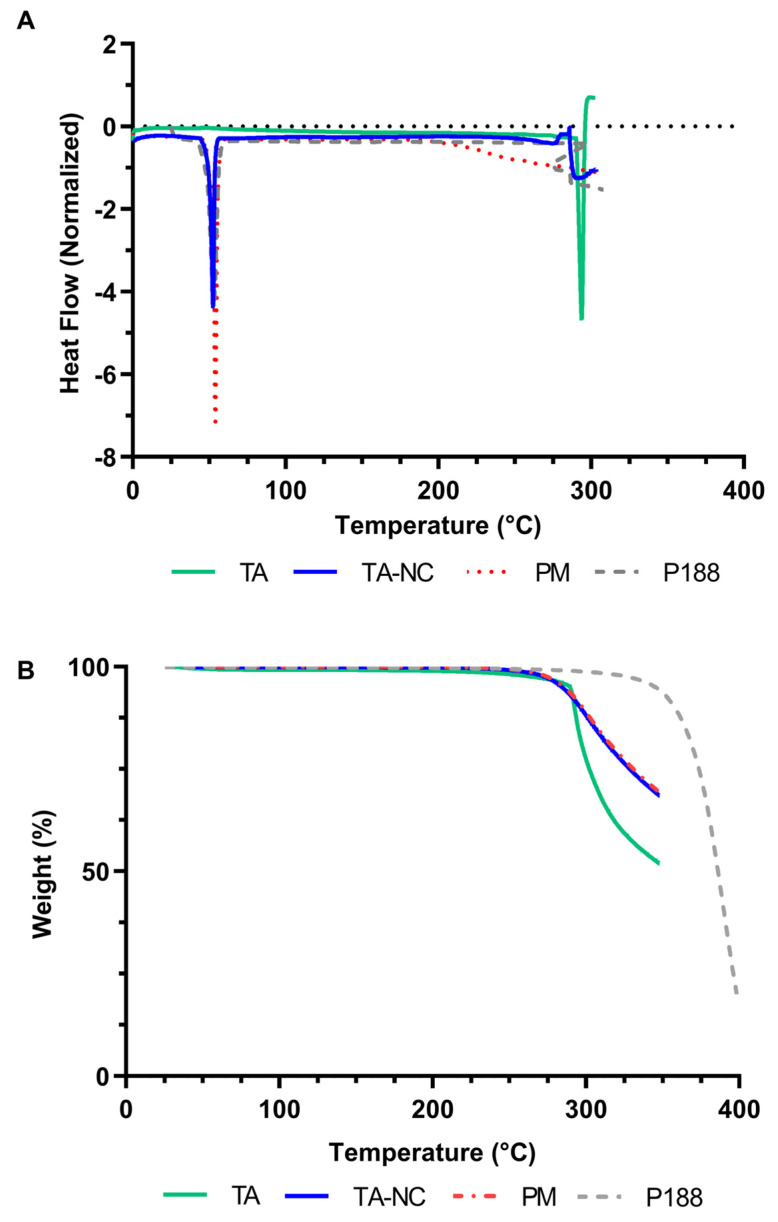
(**A**) Differential scanning calorimetry (DSC) of mTA, P188, PM and TA-NC. (**B**) Thermogravimetric analyses (TGA) of mTA, P188, PM and TA-NC.

In order to evaluate the thermal properties of TA-NC, DSC and TGA studies were carried out. As shown in Figure 6A, pure mTA presented a characteristic sharp endothermic peak at 293.9 °C associated with the melting point of this drug in the anhydrous crystalline state [23], while pure P188 exhibited a sharp endothermic melting peak at 52.9 °C. Moreover, the TGA study showed that the weight loss of all samples was negligible between 25 and 250 °C, indicating a low moisture content, as also demonstrated above (Table 1). As shown in Figure 6B, mTA exhibited more than a 50% weight loss between 260 and 350 °C, while P188 showed a high weight loss between 300 and 400 °C (80%). Comparing the DSC and TGA thermograms, the results indicate that the melting of TA occurs simultaneously with its decomposition.

In relation to TA-NC, the DSC thermograms exhibited, on the one hand, a well-defined peak at 52.6 °C corresponding to the melting point of P188 and, on the other hand, an endothermic event at 291.9 °C associated with TA, which appears as a poorly defined peak, less intense in terms of endothermic units and slightly shifted in relation to mTA. In the PM sample, endothermic events related to the melting point of the stabilizer and the drug were also observed. Both TA-NC and PM showed a mass loss after the 250 °C range in the TGA study, also evidencing the decomposition of the drug. No significant differences or new peaks associated with glass transition or recrystallization were observed between the DSC thermograms of the TA-NC and the PM.

### 3.6. X-ray Powder Diffraction (XRPD)

**Figure 7:**
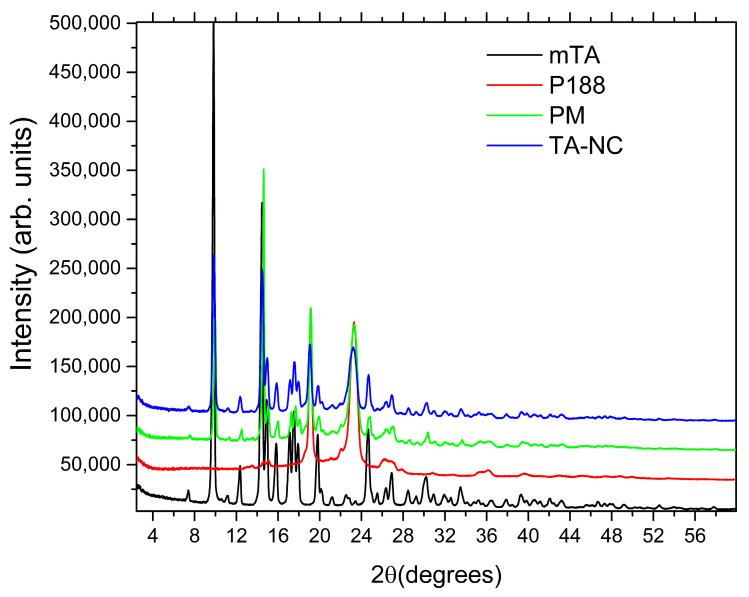
XRPD patterns for mTA, P188, PM and TA-NC.

XRPD analysis was carried out to confirm the crystalline state of dried TA-NC. XRPD patterns of mTA, P188, PM and TA-NC are illustrated in Figure 7. Pure mTA exhibited distinctive sharp peaks at 2θ angles of 9–22° and 24–32°, specifically at 9.7°, 14.4°, 17.4°, 19.7°, 24.5° and 30°, indicating the typical crystal structure of the drug [24,25]. On the other hand, P188 presented less crystalline characteristics, with only two main peaks located at 2θ angles of 19.0° and 23.1°. In relation to XRPD patterns of PM and TA-NC, the characteristic peaks of mTA were found in both samples, indicating that the drug remained in crystalline form after the preparation process. Taking into consideration these outcomes with the thermal behaviour observed by DSC, it was concluded that the crystalline structure of TA was largely preserved and neither amorphization nor the formation of polymorphs resulted after bead milling. In reference to the excipient P188, small changes occurred during the PM and TA-NC preparation, evidenced by small changes in the relative intensities of the main two peaks. Nevertheless, the crystallinity of P188 remained almost nonaltered during the preparation of both formulations.

### 3.7. Confocal Raman Microscopy

**Figure 8:**
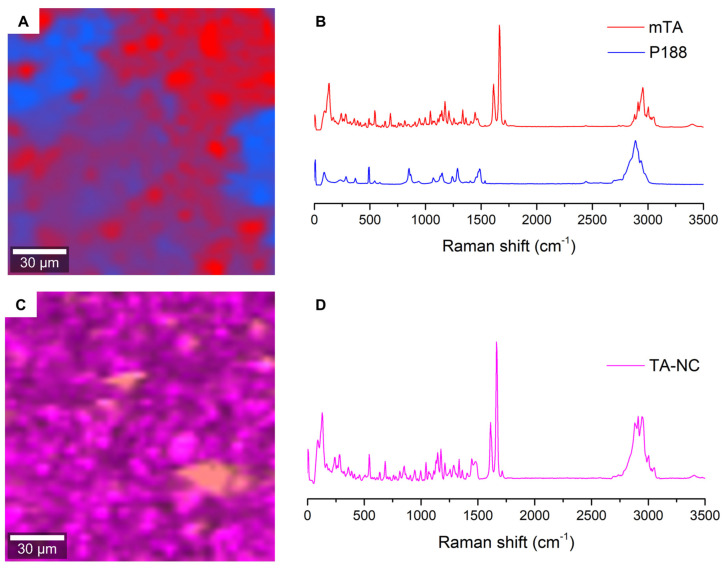
(**A**) Confocal Raman image for PM showing the distribution of the most relevant components (coinciding with mTA and P188) and (**B**) their corresponding averaged spectra according to their selected areas. (**C**) Confocal Raman image for TA-NC, showing a strong homogeneity, as evidenced in (**D**) the average Raman spectra.

Confocal Raman spectroscopy was very useful to analyze the formulations in the solid state and to evaluate the distribution of components in the TA-NC and PM samples. The Raman analysis of the pure components helped to identify characteristic Raman signals of mTA (most notably ~1670 cm^−1^) and P188 (~841 cm^−1^), both signals are important because of their intensity and negligible overlap of signals from both components. The Raman spectra of the pure components are shown in Figure 8B.

The mapping images of the PM and TA-NC samples are shown in Figure 8A,C, respectively. True component analysis (WITec, ProjectFive 5.1 Plus software) of the PM allowed us to identify two different principal components, corresponding to the drug and stabilizer. This reconstructed image (Figure 8A) shows areas where either pure P188 or TA are detected with extensions in the range of ~50 µm and some small areas where the two components are present. On the other hand, the chemical topographic mapping image of the TA-NC sample showed a highly homogeneous distribution of both TA and P188 (Figure 8C), where the spectra collected for each pixel were virtually identical and exclusive areas of the polymer or drug cannot be detected. The mean spectrum of all pixels is shown in Figure 8D. From these results, we can conclude that TA-NC is highly homogeneous, at least at the resolution of the confocal Raman microscope (~300 nm for an excitation laser operating at lambda = 532 nm).

### 3.8. Drug Saturation Concentration

**Figure 9:**
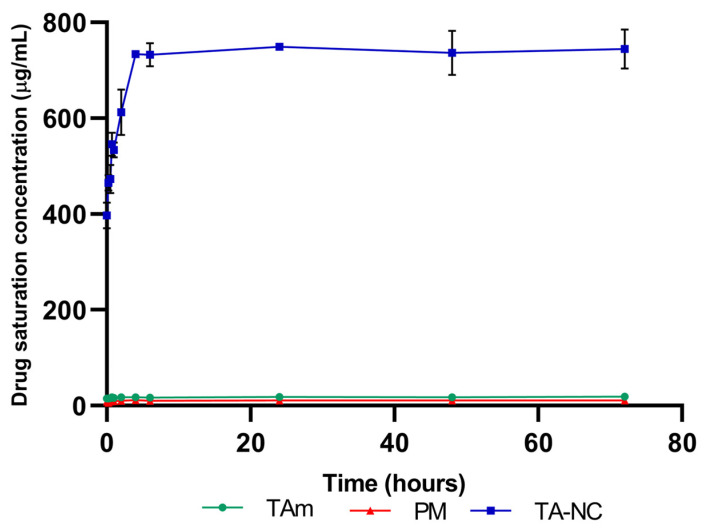
Drug saturation concentration studies of TA-NC, mTA and PM in the simulated tear fluid at 37 °C. Results are presented as means ± SD (*n* = 3).

The drug saturation concentration in aqueous media of TA-NC was performed and compared with PM and mTA to evaluate the influence of reducing the particle size on the saturation concentration of TA. The results revealed that TA-NC presented a forty-fold higher drug saturation concentration in a simulated tear fluid than mTA (Figure 9). After incubation at 37 °C, the TA-NC reached a drug saturation concentration of 745 ± 41 µg/mL, while the mTA and PM showed a drug saturation concentration of only 18 ± 1 µg/mL and 11.1 ± 0.3 µg/mL, respectively.

### 3.9. Ocular Tolerance and Irritation Test

**Figure 10:**
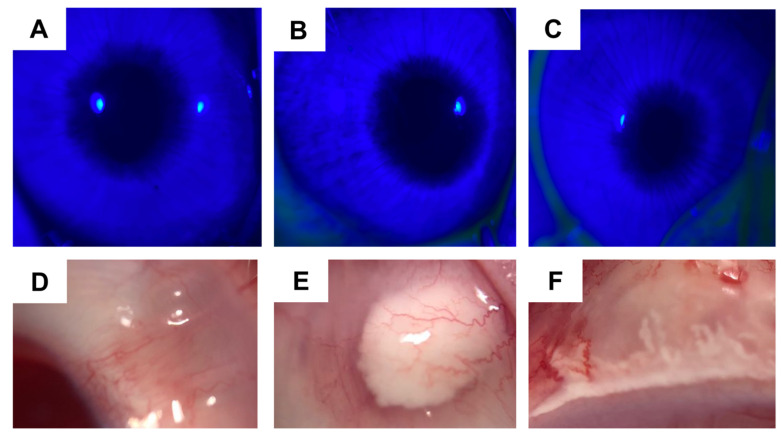
Images of fluorescein staining after 24 h of subconjunctival administration of (**A**) normal saline solution, (**B**) Fortcinolona^®^ 40 and (**C**) aqueous dispersible TA-NC to evaluate corneal damage. Images of conjunctiva rabbit eye after 24 h of subconjunctival administration of (**D**) normal saline solution, (**E**) Fortcinolona^®^ 40 and (**F**) aqueous dispersible TA-NC to evaluate conjunctiva redness.

Figure 10 shows rabbit eyes 24 h after the administration of NSS, TA-NC and Fortcinolona^®^ 40. Clinical signs after subconjunctival administration of water-dispersed TA-NC (2 mg) were examined by slit-lamp and binocular indirect ophthalmoscopy. The study revealed the absence of corneal damage, opacity, conjunctival chemosis, conjunctival redness, vitreous haze and retinal damage in all rabbits after 24 h, 48 h, 72 h and 1 week of formulation administration. No conjunctival irritation was observed in any of the groups. Comparable results were observed after subconjunctival administration of NSS and Fortcinolona^®^ 40 at a comparable dose to TA-NC. A group of normal animals was used to compare possible injection-associated alterations. In addition, no apparent lesions were observed in the cornea epithelium by fluorescein staining for either treatment (Figure 10A–C). On the other hand, a delimited deposition of the drug Fortcinolona^®^ 40 could be observed in Figure 10E, while TA-NC seems to be deposited diffusely (Figure 10F).

### 3.10. In Vivo Therapeutic Efficacy of TA-NC in EIU Model

**Figure 11:**
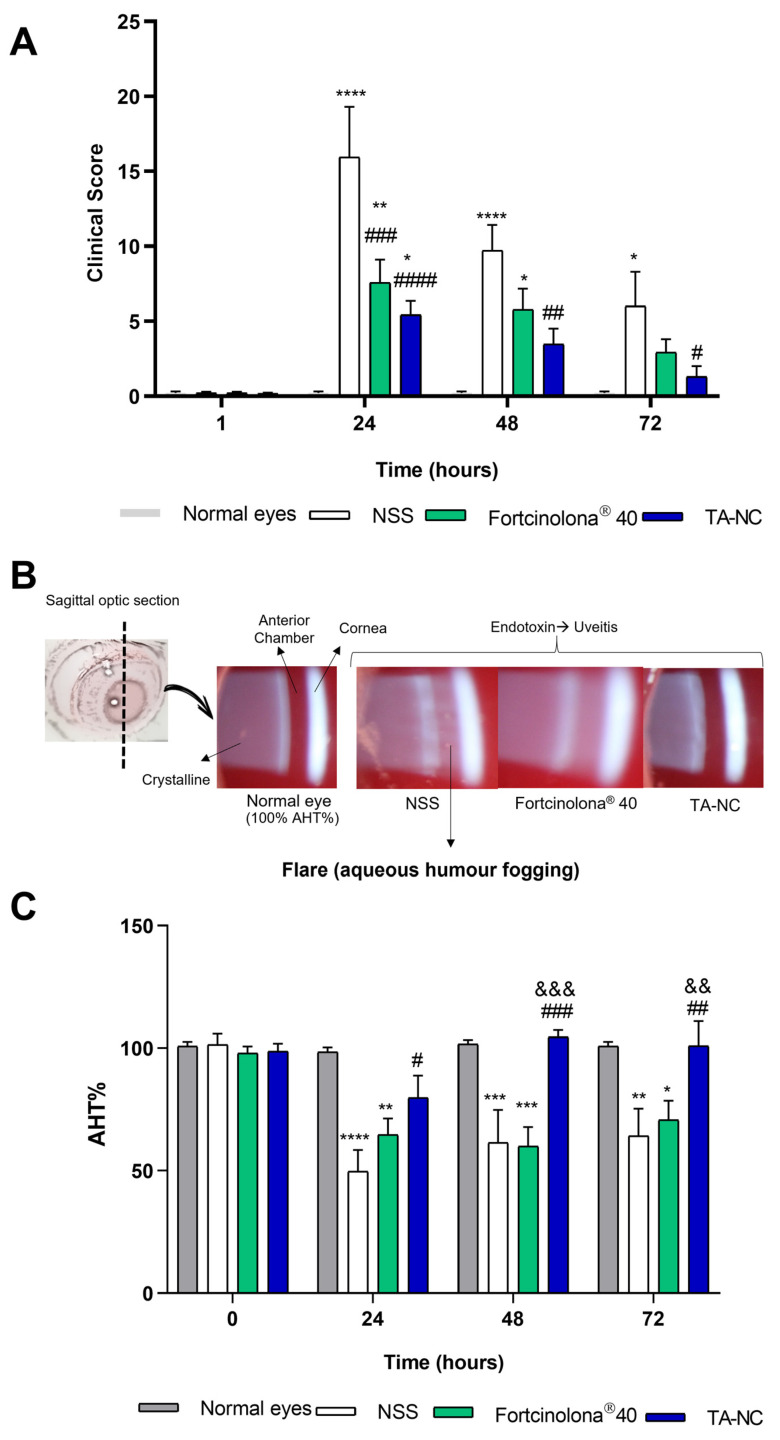
Evaluation of therapeutic efficacy of TA-NC. (**A**) Clinical inflammation score of rabbits treated with normal saline solution (NSS), TA-NC and Fortcinolona^®^ 40 after 24 h, 48 h and 72 h of EUI model in comparison to normal animals. (**B**) Representative photographs taken with slit lamp of the anterior chamber of normal eye and eyes exposed to EIU in vivo model (induced by LPS) after 24 h of subconjunctival treatment with NSS, Fortcinolona^®^ 40 and TA-NC. (**C**) Aqueous humour transparency percentage (AHT%) of the rabbit eye after 24, 48 and 72 h of EIU in vivo model and subconjunctival treatment with NSS (*n* = 8), Fortcinolona^®^ 40 (*n* = 8) and TA-NC (*n* = 8) in comparison with normal eyes (*n* = 8). AHT% was calculated from the difference in the mean grey between the corneal light reflection and the anterior chamber. Results are presented as means ± SEM for all groups. A two-way ANOVA test was used, and a significant difference was considered to be: * *p* < 0.05; ** *p* < 0.005; *** *p* < 0.001; and **** *p* < 0.0001 in relation to the group of normal eyes. The symbols # *p* < 0.05, ## *p* < 0.005, ### *p* < 0.001, #### *p* < 0.0001 and && *p* < 0.005, &&& *p* < 0.001 are used to refer to significant differences in relation to the NSS group and Fortcinolona^®^ 40 group, respectively.

The in vivo anti-inflammatory efficacy of aqueous dispersible TA-NC was assessed in an EIU rabbit model. As was described above, evaluation of clinical signs revealed maximal intraocular inflammation after 24 h of ocular application of endotoxin. Figure 11A shows the combined mean clinical inflammation score based on clinical signs for the different groups of animals. Rabbits injected with NSS showed clinical inflammatory signs such as conjunctival redness and congestion of iris vessels, protein and fibrin deposits in the cornea and crystalline lens, significant anterior chamber cells (Tyndall effect) and aqueous humour flares. Moreover, IOP decreased after model induction in comparison with baseline IOP in all groups of animals injected with LPS. In contrast, animals injected with an aqueous dispersion of TA-NC significantly alleviated the inflammatory response in the anterior chamber and iris after 24, 48, and 72 h of application compared with animals injected with NSS. Fortcinolona^®^ 40 significantly alleviated clinical inflammatory signs relative to the NSS-injected group after 24 h of model induction; however, no significant differences were observed in clinical scores between these groups after 48 and 72 h. Therefore, TA-NC proved to be the most effective anti-inflammatory treatment under the conditions evaluated.

In uveitis, aqueous humour fogging (flare) in the anterior chamber and the Tyndall effect are the main clinical signs of this ocular disorder, being directly proportional to the observed inflammation. These clinical signs are caused by protein leaking from inflamed blood vessels and cellular infiltration from inflammation of the iris and ciliary body. They can be visualized in the red pupil fondus at maximum slit lamp light intensity. As displayed in Figure 11B, in photographs obtained by a camera attached to the slit lamp, increased fogging was observed in the aqueous humour of the anterior chamber of animals exposed to LPS and injected with NSS. As a brief description of the sagittal optic section of the eye exhibited in the photographs, the cornea is on the right, the anterior chamber is in the centre and the crystalline lens is on the left. In normal eyes, the aqueous humour in the anterior chamber appears transparent, not showing any type of opacity and reflecting the normal red colour of the eye fundus. After 24 h of LPS injection, foggy aqueous humour was observed in the eyes of animals with uveitis and injected with NSS, whereas less fogginess of the aqueous humour was observed when the eyes were treated with TA-NC or Fortcinolona^®^ 40.

The degree of anterior chamber inflammation exhibited in the slit lamp images and aqueous humour fogging of the anterior chamber were analyzed by Image J software and quantified as the mean grey difference between the corneal light reflex and the anterior chamber. The calculated AHT% relative to the basal mean grey difference of the normal eye (which corresponds to 100%) is shown in Figure 11C. The significantly greater AHT% in the TA-NC-treated eye compared with NSS reveals a significant attenuation of the aqueous humour fogging after administration of the nanoparticulate formulation. In contrast, the AHT% in the Fortcinolona^®^ 40-treated eye did not show a significant difference relative to NSS. Thus, TA-NS clearly attenuated ocular inflammation in the animals after the EUI model, with a lower clinical inflammation score and achieving greater aqueous humour transparency. Moreover, TA-NC presented a superior effect relative to Fortcinolona^®^ 40, demonstrating greater therapeutic efficacy in this in vivo model of ocular inflammation.

## 4. Discussion

TA is a synthetic glucocorticoid widely used as a first-line treatment for several ocular pathologies due to its anti-inflammatory and immunomodulatory effects and low cost. Nevertheless, the TA suspensions commonly used as off-label ophthalmic medicines present certain limitations, mainly associated with their particle sizes, excipients and administration routes. The development of novel therapeutic formulations to improve the ocular efficacy of TA, especially those requiring lower doses and less frequent administration and presenting fewer drug side effects, would be extremely useful. In this regard, the design of an NC-based TA formulation with simple composition obtained by a scalable process is proposed. Thus, a self-dispersible TA-NC was developed as a therapeutic strategy for ocular application produced by WBM followed by the spray-drying process.

The WBM is a top-down method for obtaining NSs that has demonstrated certain advantages associated with the simplicity of the formulation and the ease of scaling up the process and, which is widely accepted by the pharmaceutical industry [26]. In a previous work of our research group, the preparation of NSs of poorly soluble drugs by WBM was studied by exploring different process parameters, showing that the increase in microsphere concentration, high motor speed and low solids content lead to smaller particle sizes and PDI [19]. In this work, an experimental mixture design for the preparation of TA-NS with a total solids content of 2% *w*/*v* was performed to evaluate the ratio of drug and stabilizer using high motor rotation frequencies (1600 rpm) and higher bead content (25%). After 2 h of milling, all TA-NS formulations showed a nanometric particle size of less than 350 nm with a narrow size distribution (PDI less than 0.2). A decrease in APS was observed in TA-NS with a high proportion of P188 and a low proportion of TA, which could be related to the higher availability of P188 to exert steric stabilization between drug particles due to its capacity to bind to their solid surface [27]. TA-NS with P188 concentrations between 1.000 and 1.375% *w*/*v* (NS3 and NS4, respectively) showed an APS around 230 nm and PDI = 0.12, while higher APS and PDI were observed with higher stabilizer concentration and lower drug concentration (NS5), which could be related to higher viscosity during the milling process and lower particle breakage efficiency [27]. As described, drug and stabilizer content can influence particle size reduction by media milling.

All TA-NS showed negative surface potentials, which appeared to increase in absolute values with high stabilizer concentration. The decline in zeta potential related to the increasing concentration of P188 could be attributed to the formation of a sterically stabilized polymeric layer. Although TA-NS presented ZP values around −25 mV, an adequate parameter of colloidal stability, APS and PDI were studied after the media milling process. As already described, the formation of nanosized particles creates high-energy surfaces, which can lead to aggregation and Ostwald ripening if stabilization is not at an efficient level. According to the Lifshitz−Slyozov−Wagner theory, in a system where small particles are in equilibrium with larger particles, the overall size and size distribution will increase over time [28]. Thus, a progressive increase in their APS and PDI was observed after 2 h under storage conditions at 25 °C, reaching micrometric sizes after 24 h. Therefore, it was determined that the removal of aqueous media from TA-NS by spray-drying should be performed immediately after bead milling.

Spray-drying is a one-step process that allows the obtainment of a powder from a liquid. The operating parameters were selected considering the process yield achieved for dry NC composed of P188 based on previous research [19], which has been shown to provide a “cryoprotectant effect” for low solids concentrations [29]. Thus, the spray-drying process of different TA-NS showed a process yield of up to 70%, obtaining powders with a moisture content below 2%. In relation to particle size, TA-NC with a low amount of drug and high concentration of stabilizer showed a decrease in APS and PDI after aqueous self-dispersion, remaining without significant changes for at least 24 h and presenting a slight difference (~30 nm) with respect TA-NS before spray-drying. Likewise, TA-NC with equal amounts of drug and stabilizer (1% *w*/*v*) exhibited a particle size of 260 nm and a narrow size distribution, resulting in the most useful formulation with which to continue the study since it allowed the obtainment of nanometric sizes without requiring low drug content and high stabilizer content. In this way, it would be possible to minimize the stabilizer concentration as is required in ocular pharmaceutical formulations and, at the same time, ensure complete redispersion of the powders obtained after spray-drying.

In addition, the dispersed TA-NC obtained from TA-NS with a higher total solids content (6%) composed of TA and P188 in a 1:1 ratio (NC6) presented a comparable size and PDI to TA-NC with the same composition and a lower total solids content (2%). As described, stabilization is necessary for the formation of NC as well as for long-term formulation stability during storage. The study of the colloidal stability of NC6 after aqueous self-dispersion revealed that it retains its APS, PDI and ZP at room temperature and 4 °C for at least 30 days, which is the maximum recommended usage time for all ophthalmic formulations once opened; while self-dispersible TA-NC powder remained stable in terms of these assessed parameters at least 120 days.

Concerning the physicochemical characterization of TA-NC, it was revealed that the manufacturing process did not affect the drug properties. TA-NC presented a spherical shape with smooth surfaces, as usually observed for spray-dried powders, and a homogeneous size distribution according to SEM studies, in agreement with results observed for other NCs obtained by WBM and spray-drying processes [19]. Additionally, Raman studies revealed a highly homogeneous distribution of both TA and P188 in TA-NC, which is in line with the analyses of other NCs composed of P188 and obtained by the same process [30]. In addition, no detectable chemical interactions between TA and P188 were observed by FTIR. In turn, XRPD studies revealed that the crystalline structure of TA was largely preserved and that neither amorphization nor polymorph formation occurred after obtaining the TA-NC, while no new peaks associated with glass transition or recrystallization were observed by DSC studies.

Among the main properties of NCs associated with the reduction in the drug particle size to the nanometric range, the increase in surface area is a key factor leading to a faster saturation of the dissolution layer around the particles when they are exposed to the solvent for dissolution and, consequently, to an increase in the dissolution rate, according to the Noyes–Whitney equation [31]. In turn, the increase in drug saturation concentration related to the increase in the curvature and dissolution pressure of the drug from the NC makes a more significant amount of dissolved molecules of the drug compound available [32,33]. Thus, the increased drug saturation concentration of TA-NC was demonstrated, reaching concentration values at least forty times higher in a simulated tear fluid than mTA and PM. Interestingly, TA-NC showed a higher increase in drug saturation concentration than those achieved for lyophilized TA nanosuspensions in other works [13].

Moreover, the satisfactory self-dispersion exhibited by TA-NC could contribute to achieving a drug saturation concentration faster, since particle separation allows exposure of the enlarged surface to aqueous media. In this way, a better spread of particles was observed in the subconjunctival space after subconjunctival injection of dispersed TA-NC compared to Fortcinolona^®^ 40, which forms a defined deposit in the injection site. Adequate particle dispersion could explain this satisfactory spread of particles after ocular administration of TA-NC, which, together with the low particle size, facilitated the injection of the formulation.

Regarding ocular tolerance, the self-dispersible TA-NC did not cause ocular damage after in vivo administration in rabbits, proving to be safe for subconjunctival application. In turn, TA-NC attenuated the clinical signs of the inflammatory response in the in vivo model of EIU, thereby demonstrating its therapeutic efficacy. As the results showed, TA-NC significantly alleviated the inflammatory response in the anterior chamber and iris after 24, 48 and 72 h of subconjunctival injection compared with the group administered with NSS. In turn, it did not show statistically significant differences with the normal eyes after 48 h of injection. Interestingly, the group of animals injected with the commercial TA Fortcinolona^®^ 40 exhibited a significant therapeutic effect on the clinical inflammatory signs relative to the NSS group only after 24 h of its administration and showed statistically significant differences after 24 and 48 h compared with normal eyes. Therefore, TA-NC proved to be the most effective anti-inflammatory treatment under the conditions evaluated.

As described previously, fogging of aqueous humour in the anterior chamber may clinically reflect the degree of inflammation in uveitis. In our study, a single dose of dispersed TA-NC significantly decreased the fogginess, which could be related to the low particle size, easy spread and increased drug saturation concentration. In turn, TA-NC exhibited a superior effect on fogginess attenuation than Fortcinolona^®^ 40 at the same dose, which could be explained by the higher drug saturation concentration that allows a larger amount of available dissolved molecules to exert a pharmacological effect. Similarly, the demonstrated therapeutic efficacy of TA-NC was achieved in this in vivo model by testing half the dose typically used for injection of TA nanosuspensions [34,35,36,37] such as off-label Fortcinolona^®^ 40 in the clinical treatment of ocular inflammations such as uveitis. Thus, TA-NC could result in a promising approach to administering lower doses of corticosteroids or decreasing the frequency of administration of the therapeutic scheme. Considering the side effects associated with chronic corticosteroid treatments, such as elevated intraocular pressure and cataract formation as well as those related to frequent intraocular injections, the administration of a lower effective dose of injectable TA is a key achievement. Therefore, TA-NC can be considered a promising alternative in the ocular delivery of TA with demonstrated in vivo efficacy.

## 5. Conclusions

This work addressed the design of a novel alternative for the treatment of ocular inflammatory disorders. A novel self-dispersible TA-NC was developed by bead milling followed by spray-drying, a method widely used in the pharmaceutical industry that allows the obtainment of self-dispersible powders with a narrow particle size distribution, lower moisture content and a high process yield. In turn, the self-dispersible TA-NC powder remained nanometric-sized for at least 120 days under storage conditions at room temperature, while the aqueous dispersed TA-NC for at least 30 days, the maximum recommended use time for all ophthalmic formulations once opened. Furthermore, a single subconjunctival administration of TA-NC was safe for ocular use and significantly mitigated clinical signs of inflammatory response in the in vivo model at a lower dose of TA than that typically applied in clinical practice. Therefore, self-dispersible TA-NC represents a new approach for ocular use for the treatment of inflammatory processes of various ocular disorders.

## Data Availability

Not applicable.

## References

[1] Smithen L.M., Ober M.D., Maranan L., Spaide R.F. (2004). Intravitreal Triamcinolone Acetonide and Intraocular Pressure. Am. J. Ophthalmol..

[2] Luo L., Yang J., Oh Y., Hartsock M.J., Xia S., Kim Y.-C., Ding Z., Meng T., Eberhart C.G., Ensign L.M. (2019). Controlled Release of Corticosteroid with Biodegradable Nanoparticles for Treating Experimental Autoimmune Uveitis. J. Control. Release.

[3] Le Merdy M., Fan J., Bolger M.B., Lukacova V., Spires J., Tsakalozou E., Patel V., Xu L., Stewart S., Chockalingam A. (2019). Application of Mechanistic Ocular Absorption Modeling and Simulation to Understand the Impact of Formulation Properties on Ophthalmic Bioavailability in Rabbits: A Case Study Using Dexamethasone Suspension. AAPS J..

[4] Wang Y., Friedrichs U., Eichler W., Hoffmann S., Wiedemann P. (2002). Inhibitory Effects of Triamcinolone Acetonide on BFGF-Induced Migration and Tube Formation in Choroidal Microvascular Endothelial Cells. Graefe’s Arch. Clin. Exp. Ophthalmol..

[5] Hirani A., Grover A., Lee Y.W., Pathak Y., Sutariya V. (2016). Triamcinolone Acetonide Nanoparticles Incorporated in Thermoreversible Gels for Age-Related Macular Degeneration. Pharm. Dev. Technol..

[6] Li J., Cheng T., Tian Q., Cheng Y., Zhao L., Zhang X., Qu Y. (2019). A More Efficient Ocular Delivery System of Triamcinolone Acetonide as Eye Drop to the Posterior Segment of the Eye. Drug Deliv..

[7] Mahran A., Ismail S., Allam A.A. (2021). Development of Triamcinolone Acetonide-Loaded Microemulsion as a Prospective Ophthalmic Delivery System for Treatment of Uveitis: In Vitro and In Vivo Evaluation. Pharmaceutics.

[8] Sarao V., Veritti D., Boscia F., Lanzetta P. (2014). Intravitreal Steroids for the Treatment of Retinal Diseases. Sci. World J..

[9] Yang Y., Bailey C., Loewenstein A., Massin P. (2015). Intravitreal Corticosteroids in Diabetic Macular Edema: Pharmacokinetik Considerations. Retina.

[10] Altamirano-Vallejo J.C., Navarro-Partida J., Gonzalez-De la Rosa A., Hsiao J.H., Olguín-Gutierrez J.S., Gonzalez-Villegas A.C., Keller B.C., Bouzo-Lopez L., Santos A. (2018). Characterization and Pharmacokinetics of Triamcinolone Acetonide-Loaded Liposomes Topical Formulations for Vitreoretinal Drug Delivery. J. Ocul. Pharmacol. Ther..

[11] Formica M.L., Gamboa G.U., Tártara L.I., Luna J.D., Benoit J.P., Palma S.D. (2020). Triamcinolone Acetonide-Loaded Lipid Nanocapsules for Ophthalmic Applications. Int. J. Pharm..

[12] Araújo J., Garcia M.L., Mallandrich M., Souto E.B., Calpena A.C. (2012). Release Profile and Transscleral Permeation of Triamcinolone Acetonide Loaded Nanostructured Lipid Carriers (TA-NLC): In Vitro and Ex Vivo Studies. Nanomedicine.

[13] Wu Y., Vora L.K., Mishra D., Adrianto M.F., Gade S., Paredes A.J., Donnelly R.F., Singh T.R.R. (2022). Nanosuspension-Loaded Dissolving Bilayer Microneedles for Hydrophobic Drug Delivery to the Posterior Segment of the Eye. Biomater. Adv..

[14] Fernandes A.R., Vidal L.B., Sánchez-López E., dos Santos T., Granja P.L., Silva A.M., Garcia M.L., Souto E.B. (2022). Customized Cationic Nanoemulsions Loading Triamcinolone Acetonide for Corneal Neovascularization Secondary to Inflammatory Processes. Int. J. Pharm..

[15] Müller R.H., Keck C.M. (2012). Twenty Years of Drug Nanocrystals: Where Are We, and Where Do We Go?. Eur. J. Pharm. Biopharm..

[16] da S., de Jesus J.I.S., Lourenço F.R., Ishida K., Barreto T.L., Avino V.C., dos S., Neto E., Bou-Chacra N.A. (2022). Besifloxacin Nanocrystal: Towards an Innovative Ophthalmic Preparation. Pharmaceutics.

[17] Chen M.-L., John M., Lee S.L., Tyner K.M. (2017). Development Considerations for Nanocrystal Drug Products. AAPS J..

[18] Gao L., Liu G., Ma J., Wang X., Zhou L., Li X. (2012). Drug Nanocrystals: In Vivo Performances. J. Control. Release.

[19] Paredes A.J., Camacho N.M., Schofs L., Dib A., del Pilar Zarazaga M., Litterio N., Allemandi D.A., Sánchez Bruni S., Lanusse C., Palma S.D. (2020). Ricobendazole Nanocrystals Obtained by Media Milling and Spray Drying: Pharmacokinetic Comparison with the Micronized Form of the Drug. Int. J. Pharm..

[20] McGuckin M.B., Wang J., Ghanma R., Qin N., Palma S.D., Donnelly R.F., Paredes A.J. (2022). Nanocrystals as a Master Key to Deliver Hydrophobic Drugs via Multiple Administration Routes. J. Control. Release.

[21] Huang J., Yu X., Zhou Y., Zhang R., Song Q., Wang Q., Li X. (2018). Directing the Nanoparticle Formation by the Combination with Small Molecular Assembly and Polymeric Assembly for Topical Suppression of Ocular Inflammation. Int. J. Pharm..

[22] Altinsoy A., Dilekoz E., Kul O., Ilhan S.O., Tunccan O.G., Seven I., Bagriacik E.U., Sarioglu Y., Or M., Ercan Z.S. (2011). A Cannabinoid Ligand, Anandamide, Exacerbates Endotoxin-Induced Uveitis in Rabbits. J. Ocul. Pharm..

[23] García-Millán E., Quintáns-Carballo M., Otero-Espinar F.J. (2017). Solid-State Characterization of Triamcinolone Acetonide Nanosuspensiones by X-Ray Spectroscopy, ATR Fourier Transforms Infrared Spectroscopy and Differential Scanning Calorimetry Analysis. Data Brief.

[24] García-Millán E., Quintáns-Carballo M., Otero-Espinar F.J. (2017). Improved Release of Triamcinolone Acetonide from Medicated Soft Contact Lenses Loaded with Drug Nanosuspensions. Int. J. Pharm..

[25] Sabzevari A., Adibkia K., Hashemi H., Hedayatfar A., Mohsenzadeh N., Atyabi F., Ghahremani M.H., Dinarvand R. (2013). Polymeric Triamcinolone Acetonide Nanoparticles as a New Alternative in the Treatment of Uveitis: In Vitro and in Vivo Studies. Eur. J. Pharm. Biopharm..

[26] Mohammad I.S., Hu H., Yin L., He W. (2019). Drug Nanocrystals: Fabrication Methods and Promising Therapeutic Applications. Int. J. Pharm..

[27] Tuomela A., Hirvonen J., Peltonen L. (2016). Stabilizing Agents for Drug Nanocrystals: Effect on Bioavailability. Pharmaceutics.

[28] Dagtepe P., Chikan V. (2010). Quantized Ostwald Ripening of Colloidal Nanoparticles. J. Phys. Chem. C.

[29] Paredes A.J., Llabot J.M., Sánchez Bruni S., Allemandi D., Palma S.D. (2016). Self-Dispersible Nanocrystals of Albendazole Produced by High Pressure Homogenization and Spray-Drying. Drug Dev. Ind. Pharm..

[30] Melian M.E., Paredes A., Munguía B., Colobbio M., Ramos J.C., Teixeira R., Manta E., Palma S., Faccio R., Domínguez L. (2020). Nanocrystals of Novel Valerolactam-Fenbendazole Hybrid with Improved in Vitro Dissolution Performance. AAPS PharmSciTech.

[31] Noyes A.A., Whitney W.R. (1897). The Rate of Solution of Solid Substances in Their Own Solutions. J. Am. Chem. Soc..

[32] Real D., Formica M.L., Picchio M., Paredes A.J., Shahzad Y., Rizvi S.A.A., Yousaf A.M., Hussain T. (2022). Manufacturing Techniques for Nanoparticles in Drug Delivery. Drug Delivery Using Nanomaterials.

[33] Van Eerdenbrugh B., Vermant J., Martens J.A., Froyen L., Humbeeck J.V., van den Mooter G., Augustijns P. (2010). Solubility Increases Associated with Crystalline Drug Nanoparticles: Methodologies and Significance. Mol. Pharm..

[34] Karasu B., Kesim E., Kaskal M., Celebi A.R.C. (2022). Efficacy of Topical Dexamethasone Eye Drops in Preventing Ocular Inflammation and Cystoid Macular Edema Following Uncomplicated Cataract Surgery with or without Injection of a Single Dose Perioperative Subtenon Triamcinolone Acetonide. Cutan. Ocul. Toxicol..

[35] Hanif J., Iqbal K., Perveen F., Arif A., Iqbal R.N., Jameel F., Hanif K., Seemab A., Khan A.Y., Ahmed M. (2021). Safety and Efficacy of Suprachoroidal Injection of Triamcinolone in Treating Macular Edema Secondary to Noninfectious Uveitis. Cureus.

[36] Kim K.W., Kusuhara S., Tachihara M., Mimura C., Matsumiya W., Nakamura M. (2021). A Case of Panuveitis and Retinal Vasculitis Associated with Pembrolizumab Therapy for Metastatic Lung Cancer. Am. J. Ophthalmol. Case Rep..

[37] Gaballa S.A., Kompella U.B., Elgarhy O., Alqahtani A.M., Pierscionek B., Alany R.G., Abdelkader H. (2021). Corticosteroids in Ophthalmology: Drug Delivery Innovations, Pharmacology, Clinical Applications, and Future Perspectives. Drug Deliv. Transl. Res..

